# Involvement of type VI secretion system in secretion of iron chelator pyoverdine in *Pseudomonas taiwanensis*

**DOI:** 10.1038/srep32950

**Published:** 2016-09-08

**Authors:** Wen-Jen Chen, Tzu-Yen Kuo, Feng-Chia Hsieh, Pi-Yu Chen, Chang-Sheng Wang, Yu-Ling Shih, Ying-Mi Lai, Je-Ruei Liu, Yu-Liang Yang, Ming-Che Shih

**Affiliations:** 1 Agricultural Biotechnology Research Center, Academia Sinica, Taipei, 11529, Taiwan; 2 Institute of Biotechnology, National Taiwan University, Taipei, 10617, Taiwan; 3 Biopesticide Division, Taiwan Agricultural Chemicals and Toxic Substances Research Institute, Council of Agriculture, Taichung, 41358, Taiwan; 4 Department of Agronomy, National Chung Hsing University, Taichung, 40227, Taiwan; 5 Institute of Biological Chemistry, Academia Sinica, Taipei, 11529, Taiwan

## Abstract

Rice bacterial blight caused by *Xanthomonas oryzae* pv. *oryzae (Xoo*) is one of the most destructive rice diseases worldwide. Therefore, in addition to breeding disease-resistant rice cultivars, it is desirable to develop effective biocontrol agents against *Xoo*. Here, we report that a soil bacterium *Pseudomonas taiwanensis* displayed strong antagonistic activity against *Xoo*. Using matrix-assisted laser desorption/ionization imaging mass spectrometry, we identified an iron chelator, pyoverdine, secreted by *P. taiwanensis* that could inhibit the growth of *Xoo*. Through Tn5 mutagenesis of *P. taiwanensis*, we showed that mutations in genes that encode components of the type VI secretion system (T6SS) as well as biosynthesis and maturation of pyoverdine resulted in reduced toxicity against *Xoo*. Our results indicated that T6SS is involved in the secretion of endogenous pyoverdine. Mutations in T6SS component genes affected the secretion of mature pyoverdine from the periplasmic space into the extracellular medium after pyoverdine precursor is transferred to the periplasm by the inner membrane transporter PvdE. In addition, we also showed that other export systems, i.e., the PvdRT-OpmQ and MexAB-OprM efflux systems (for which there have been previous suggestions of involvement) and the type II secretion system (T2SS), are not involved in pyoverdine secretion.

Iron is an essential element for all living organisms. Iron is a cofactor for many enzymes, such as those that participate in critical biological processes such as redox chemistry, the tricarboxylic acid cycle, electron transport, and DNA and RNA synthesis^1^,^2^,^3^,^4^. In aerobic environments with near neutral pH, iron is insoluble and not bioavailable. As a result bacteria, fungi and graminaceous plants need to secrete iron-chelating molecules such as siderophores^5^ to scavenge iron from the environment. Fluorescent pseudomonads secrete peptidic siderophores called pyoverdines which are the primary siderophores of *Pseudomonas* bacteria^6^.

Pyoverdines are peptide-derived molecules, assembled by nonribosomal peptide-synthetase (NRPS) enzymes^7^. Pyoverdines contain variant peptide side chains with each pyoverdine having a different composition of amino acids, and a conserved fluorescent chromophore^7^,^8^. To date, more than 60 pyoverdines with diverse structures have been found in different *Pseudomonas* species, such as plant growth-promoting *P. fluorescens* SBW25, plant pathogen *P. syringae*, and human pathogen *P. aeruginosa*^9^,^10^,^11^,^12^. Pyoverdines have a strong binding affinity with ferric ion (Fe^3+^), which allows *Pseudomonas* to colonize a wide range of ecological niches^13^.

The siderophore uptake system has been extensively studied in bacteria, but its efflux system is not understood very well. So far only the MexAB-OprM and PvdRT-OpmQ efflux systems have been suggested to be involved in newly synthesized pyoverdine secretion^14^,^15^. However, Imperi *et al*. obtained results that contrasted with these reports^16^.

In this study, we re-explored the efflux systems that might be involved in the secretion of endogenous pyoverdine, and report the antagonistic activity of entomopathogenic bacterium *Pseudomonas taiwanensis*^17^ against rice blight pathogen *Xanthomonas oryzae* pv. *oryzae (Xoo*). We used MALDI-imaging mass (MALDI-IMS) to detect small molecular weight compounds secreted from wild-type and Tn-5 transposon-tagged mutants. Our results indicate that a low molecular weight pyoverdine (*m/z* 1044) is responsible for the anti-*Xoo* activity. Interestingly, we found that the type VI secretion system (T6SS) is involved in the secretion of this pyoverdine. T6SS is a protein secretion system that is widespread in Gram-negative bacteria. It can directly inject toxins into the target bacterial cells. For example, T6SS of *P. aeruginosa* and *Serratia marcescens* can target bacterial cells and induce strong toxicity with toxic proteins Tse2 and VgrG, respectively^18^,^19^. However, it has not been reported that T6SS is involved in the secretion of small molecules or peptides. It is unclear whether T6SS can be involved in secondary metabolite secretion or translocation. Our results indicate that the low molecular weight pyoverdine (*m/z* 1044) is responsible for the anti-*Xoo* activity. T6SS mutants have reduced secreted mature pyoverdine suggesting the participation of alternative pathways in the export of mature pyoverdine. Further, our study excludes the MexAB-OprM and PvdRT-OpmQ efflux systems, and protein type II secretion system in the export of endongeous mature pyoverdine.

## Results

### Anti-Xoo activity

**Figure 1:**
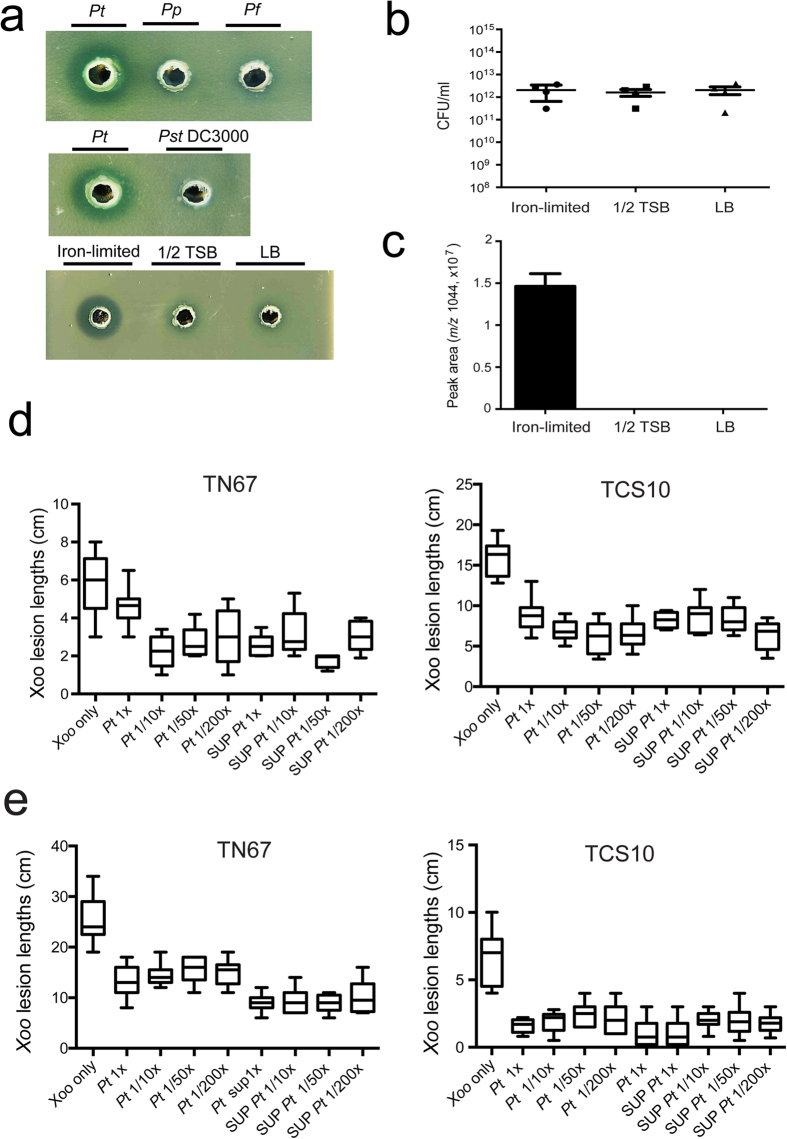
Anti-*Xoo* activity on plate assays and in field trials. (**a**) Comparison of the toxicity of *P. taiwanensis (Pt*)*, P. putida (Pp*), P. *fluorescens (Pf)*, and *Pseudomonas syringae pv. tomato* DC3000 (*Pst* DC3000) was performed on a *Xoo*-containing 1/2 TSB agar plate (top A). Bacteria were precultured in iron-limited media for 24 h, and then tested in antagonistic assay against *Xoo* with OD_600_ of 0.5. Three culture media (iron-limited, 1/2 TSB, and LB) were tested for toxicity to *Xoo* (bottom a). (**b**) Cell numbers of *P. taiwanensis* were not affected by incubation with iron-limited, 1/2 TSB, or LB media. (**c**) Comparison of the cell free culture supernatant of factor (*m/z* 1044) by LC-MS in 3 different media (iron-limited, 1/2 TSB, LB) after 24 h incubation. (**d**,**e**) Field trials of the suppression of rice blight disease by *P. taiwanensis.* Japonica rice Tainung 67 (TN 67) and indica rice Taichung Sen 10 (TCS 10) were inoculated with total broth and cell-free supernatant of *P. taiwanensis* after Xoo infection. Total broth and cell-free supernatant were made in different dilutions from 10-fold to 200-fold. Field trials were conducted in two seasons. (**d**) The first field trial was performed in May 2015. (**e**) The second field trial was performed in October 2015.

We screened several *Pseudomonas* species to search for potential biocontrol agents against *Xoo*. Bacteria were cultured in iron-limited media for 24 h, and then used in antagonistic assay against *Xoo* with OD_600_ of 0.5. One of these microbes, *P. taiwanensis,* displayed strong antagonistic activity against *Xoo*, while *P. putida* and *P. fluorescens* and plant pathogen *P. syringae* pv*. tomato* DC3000 did not induce any antagonistic activity against *Xoo* (Fig. 1a, top panel)*. P. taiwanensis* is a Gram-negative bacterium isolated from soil that can grow on a medium with shrimp shell powder as the sole carbon and nitrogen source^19^. *P. taiwanensis* was precultured in three culture media (iron-limited, 1/2 TSB, and LB) to test for toxicity to *Xoo* (Fig. 1a, bottom panel). *P. taiwanensis* showed strongest anti-*Xoo* activity when it was grown on iron-limited medium (Fig. 1a, bottom panel). When grown on nutrient rich media (LB and 1/2 TSB) or iron-limited medium, it had similar growth rates (Fig. 1b). This suggests that the anti-Xoo activity of *P. taiwanensis* under iron-limited conditions is not due to an increase in the growth rate. Interestingly, we found that a compound with *m/z* 1044 could be detected at a higher level in the iron-limited medium compared to the nutrient rich media (1/2 TSB and LB) using LC-MS detection (Fig. 1c). In order to determine whether *P. taiwanensis* could control *Xoo*-induced leaf blight in rice after incubation in iron-limited medium, we conducted field trials using the japonica rice Tainung 67 (TN 67) and indica rice Taichung Sen 10 (TCS 10) in May and October 2015 (Fig. 1d,e). TN 67 and TCS 10 were sprayed with total broth and cell-free supernatants of *P. taiwanensis* after *Xoo* infection. Total broth and cell-free supernatants were made in different dilutions from 10-fold to 200-fold. The results of the field trials showed that total broth and cell-free supernatants of *P. taiwanensis* significantly decreased the disease symptoms caused by *Xoo*.

### Pyoverdine is a toxin factor and T6SS is necessary for the secretion of pyoverdine

**Figure 2:**
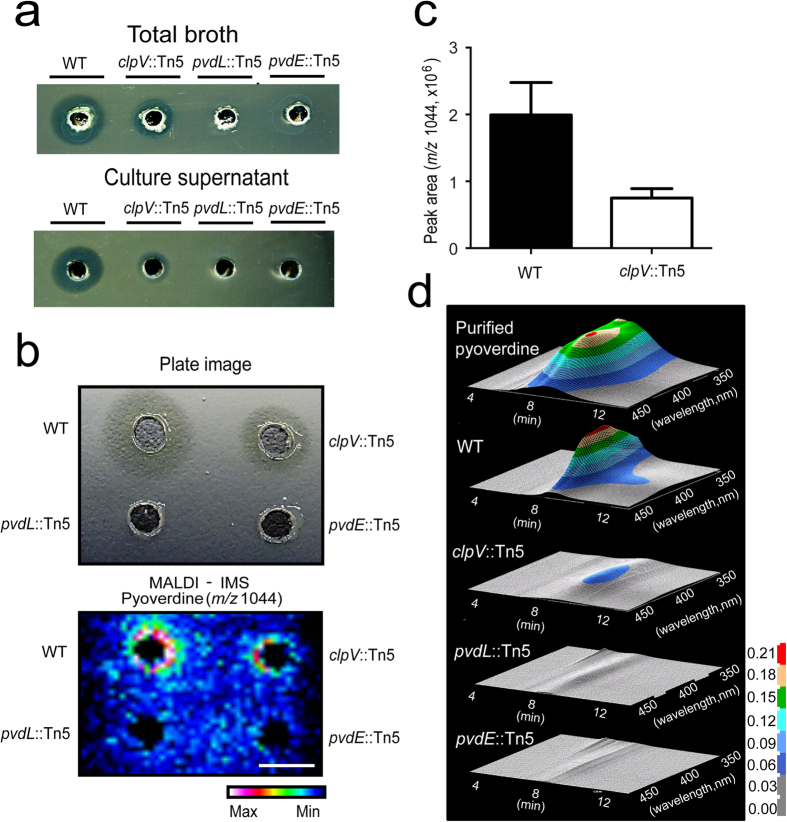
Characterization of the pyoverdine secretion system. (**a**) Zone of inhibition assay for anti-*Xoo* activity was tested in 1/2 TSB medium which contained *Xoo*. Whole culture (top a) and cell-free culture supernatant of WT, *clpV*::Tn5, *pvdL*::Tn5, and *pvdE*::Tn5, which were collected after incubation for 24 h in LP media, were injected into the hole to test toxicity against *Xoo* (bottom a). (**b**) MALDI**-**IMS imaging of pyoverdine from incubation of wild-type and mutant *P. taiwanensis* with *Xoo*. Competition plates of *P. taiwanensis* and mutants co-cultured with *Xoo* for IMS (top b). The MALDI-IMS image shows an ion of *m/z* 1044 [M + H]^+^, displaying the highest level surrounding the hole between the wild-type and mutant treatment (bottom b). Scale bar, 2 mm. Intensity gradients for pyoverdine are illustrated by color histograms (maximum, white; minimum, black). (**c**) LC-MS quantification of pyoverdine in cell free culture supernatants of WT and *clpV*::Tn5 after 24-h incubation in iron-limited media. (**d**) HPLC quantification of pyoverdine (*m/z* 1044) and culture supernatant from wild-type and mutants. Fluorescent pyoverdine was monitored at 400 nm under the UV detector. Pyoverdine was purified using Cu-sepharose.

To identify the factors that affect the antagonistic activity of *P. taiwanensis* against *Xoo*, we generated Tn5 transposon-insertion mutant pools of *P. taiwanensis* and screened for mutants with attenuated antagonistic activities against *Xoo*. A total of 5000 colonies screened, three mutants were isolated and the location of Tn5 insertion was determined using thermal asymmetric interlaced polymerase chain reaction (TAIL-PCR) and sequencing analyses to have Tn5 insertion sites in genes that encode T6SS (*clpV*, GenBank AC no. KM061430), pyoverdine synthetase (*pvdL*, GenBank AC no. KM036007) and pyoverdine translocation and maturation (*pvdE*, GenBank AC no. KM036029). ATPase ClpV is an important core component of T6SS and contributes to VipA/VipB tubule remodeling, which is required for T6SS activity^20^. PvdL is a nonribosomal peptide synthetase (NRPS) that is involved in the biosynthesis of pyoverdine chromophore^8^. PvdE is a cell membrane protein that is involved in transportation of pyoverdine precursors to the periplasm^21^. In antagonistic assays, whole culture or cell-free culture supernatants of wild-type *P. taiwanensis* showed substantial toxicity towards *Xoo* (Fig. 2a) suggesting that soluble compounds were secreted by *P. taiwanensis* to inhibit the growth of *Xoo*. In contrast, whole culture or cell-free culture supernatants of *clpV*::Tn5 mutant showed a lower toxicity than those of the wild-type, and those of *pvdL*::Tn5 and *pvdE*::Tn5 mutants exhibited no toxicity towards *Xoo*. No significant differences in the growth rates of the wild-type (WT), *clpV*::Tn5, *pvdL*::Tn5 and *pvdE*::Tn5 mutants were detected from 4 h (lag phase) to 72 h (stationary phase) under iron-limited LP broth cultures (Supplementary Fig. S1), indicating that the reduction of anti-*Xoo* activities of these mutants is not due to reduced viability.

In antagonistic assays, a signal of much lower intensity at *m/z* 1044 was detected for *clpV*::Tn5 mutant, and no signal at *m/z* 1044 was detected for *pvdL*::Tn5 and *pvdE*::Tn5 mutants (bottom panel, Fig. 2b), suggesting that *m/z* 1044 corresponds to a pyoverdine analogue. To characterize the role of T6SS in pyoverdine secretion, we used IMS to detect the pyoverdine secreted in iron-limited agar plates of the *clpV*::Tn5 mutant and the wild-type (Supplementary Fig. S2). The amount of pyoverdine in the *clpV*::Tn5 mutant was much lower than that in the wild-type cultured on the surface and cross section of iron-limited agar plates (Supplementary Fig. S2a and S2b). *P. taiwanensis* secretes siderophore pyoverdine under iron-limited conditions; in addition, pyoverdine production is not stimulated by the competitor (*Xoo*) (Supplementary Fig. S3).

Quantification using LC-MS showed that the pyoverdine level in wild-type is about 2-fold higher than in the *clpV*::Tn5 mutant (Fig. 2c). The pyoverdine (*m/z* 1044) secreted by *P. taiwanensis* was further purified using a Cu-sepharose column followed by MALDI MS monitoring (Supplementary Fig. S2). The fluorescent pyoverdine with the strongest absorbance at 400 nm was monitored by a UV detector in HPLC analysis^22^ (Fig. 2d). The supernatants from the cultures of the *clpV*::Tn5 mutant had a lower concentration of pyoverdine than that of the wild-type. In addition, we did not detect pyoverdine in the culture supernatants of the *pvdL*::Tn5 mutant (Fig. 2d), which is consistent with the results from MALDI-IMS.

### Structural characterization of pyoverdine through the secretion of *P. taiwanensis*

**Figure 3:**
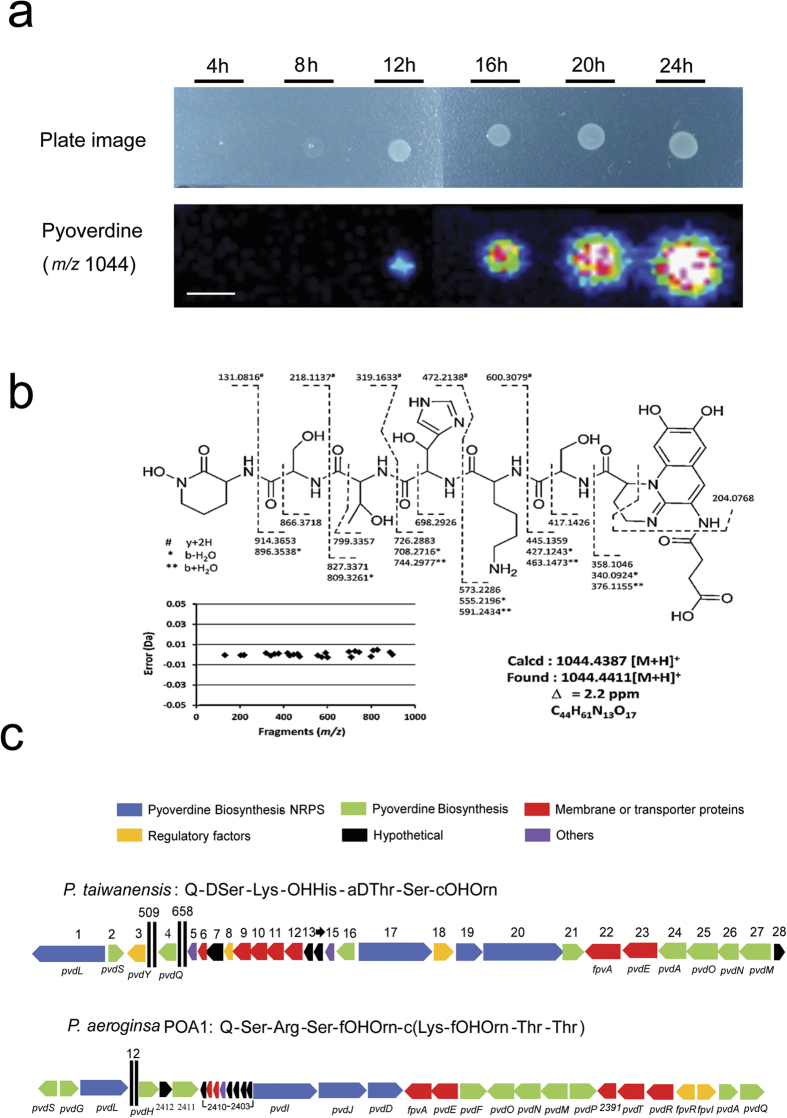
Identification of toxin factor pyoverdine from *P. taiwanensis*. (**a**) Time course of *P. taiwanensis* cultured for MALDI-IMS to detect *m/z* 1044 signal after incubation on the iron-limited agar plates for 4, 8, 12, 16, 20, and 24 hours. Scale bar, 10 mm. (**b**) Structure of pyoverdine and characteristic ions in the ESI-Orbitrap. (**c**) Comparison of the pyoverdine loci of *P. taiwanensis* and *P. aeroginsa* POA1. Genes in the pyoverdine loci of *P. taiwanensis* were annotated based on the sequences in the *Pseudomonas* genome database. The genes are classified into colors according to their functions. The arrangement of genes of the pyoverdine loci in *P. aeroginsa* POA1 according to Ravel and Cornelis^43^ was confirmed by the *Pseudomonas* Genome database. The orthologous genes in *P. taiwanensis* were assigned a number and are shown in Supplemental Table S2.

We used matrix-assisted laser desorption/ionization imaging mass spectrometry (MALDI-IMS) to survey the metabolites secreted from *P. taiwanensis* on the surface of iron-limited agar plates (Fig. 3a). Using MALDI-IMS, the spatial distribution of molecules can be visualized without labeling^23^. Plates with wild-type *P. taiwanensis* gave a pyoverdine signal at *m/z* 1044. Under iron-limited conditions, the *m/z* 1044 signal of pyoverdine was found around *P. taiwanensis* colonies after 12-h incubation in time course experiments (Fig. 3a). To determine whether this compound affects the antagonistic activity of *P. taiwanensis* against *Xoo*, we purified *m/z* 1044 compound of pyoverdine using a Cu-sepharose column (Supplementary Fig. S4) and identified its structure (Fig. 3b). The structure of the *m/z* 1044 signal was elucidated by tandem mass spectrometry (Fig. 3b, Supplementary Fig. S5a, 5b, and Supplementary table S1). The amino acid sequence deduced by tandem mass fragmentation analysis corresponded to gene sequence analysis of NRPS adenylation domain specificity (Ser-Lys and Thr-Ser-OH-Orn). The biosynthesis and transport of the pyoverdines have been studied extensively in *Pseudomonas aeruginosa* PAOl 7,24. The majority of pyoverdine biosynthetic and transport genes form a cluster in both *P. taiwanensis* and *P. aeruginosa* PAOl, whereas the *pvdL* gene is located in a separate cluster in both species (Fig. 3c).

### Complementation assay

**Figure 4:**
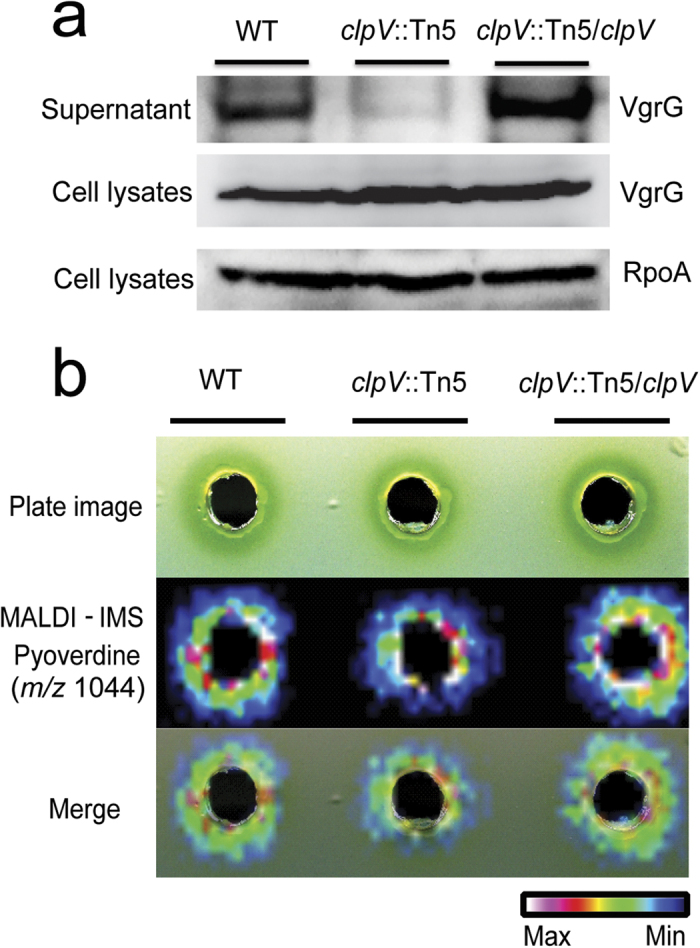
Complementation assay. (**a**) ClpV is an essential component of T6SS. T6SS activity was detected by western blotting using VgrG antibody from culture supernatants and cell lysates of wild-type, *clpV*::Tn5 and *clpV*::Tn5*/clpV*. The level of RpoA, which was used as a loading control, was detected by western blotting in cell lysates with a specific antibody. (**b**) Overexpression of ClpV in *clpV*::Tn5 mutant is able to complement pyoverdine secretion. The complemented vector pCPP30 harboring a *clpV* fragment was analyzed for pyoverdine secretion in the *clpV*::Tn5 mutant by MALDI-IMS. MALDI**-**IMS imaging of pyoverdine from incubation of wild-type, *clpV*::Tn5 mutant and *clpV*::Tn5*/clpV P. taiwanensis* with *Xoo* were monitored.

To ensure that the mutation in *clpV* affected T6SS activity in *P. taiwanensis,* two experiments were performed. First, western blot analysis was used to quantify the levels of VgrG protein, which is a biomarker for T6SS activity^25^,^26^,^27^, in cell-free culture supernatants. The results showed that VgrG could be detected in cell-free culture supernatants of wild-type and *clpV* complemented stain *clpV*::Tn5*/clpV* (Fig. 4a, top panel). In contrast, no significant level of VgrG could be detected in the culture supernatant of *clpV*::Tn5 mutant. The results also showed that the levels of VgrG in the cell lysates are similar for wild-type, *clpV*::Tn5 and *clpV*::Tn5*/clpV* (Fig. 4a, middle panel). RNA polymerase α-subunit RpoA was used as a loading control (Fig. 4a, bottom panel). These results indicate that in *P. taiwanensis* T6SS is involved in anti-*Xoo* activity by secreting pyoverdine into the medium.

Second, the *clpV*::Tn5 mutant harboring a complementary vector pCPP30 containing a *clpV* fragment was analyzed for pyoverdine secretion by MALDI-IMS. In the MALDI-IMS assays, the introduction of wild-type *clpV* into *clpV*::Tn5 mutant restored the secreted level of pyoverdine in the culture plate (Fig. 4b).This result indicates that the reduced secretion of pyoverdine in the *clpV*::Tn5 mutant results from a mutation in the *clpV* locus.

### Pyoverdine was accumulated in the periplasm of T6SS mutants

**Figure 5:**
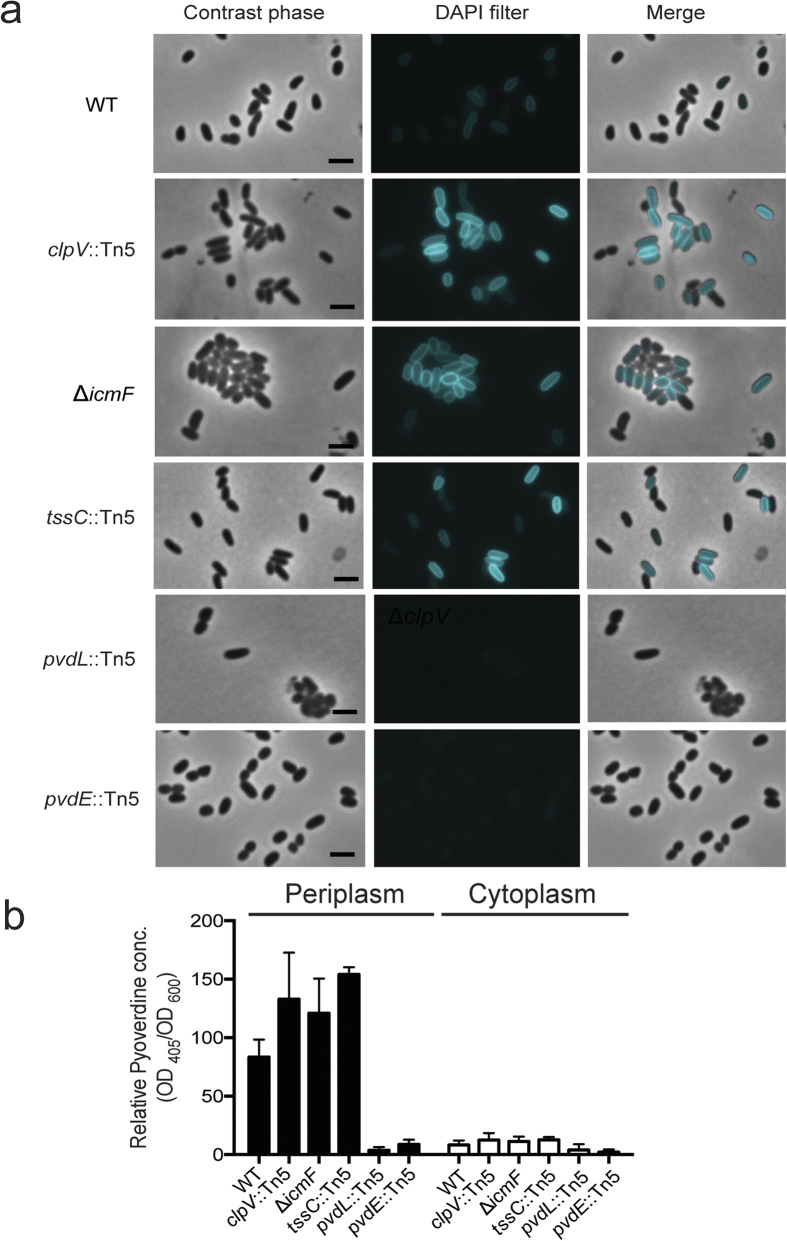
Subcellular localization and quantification of mature fluorescence pyoverdine in the periplasm and cytoplasm. (**a**) Localization of mature fluorescence pyoverdine was detected in wild-type, *clpV*::Tn5, Δ*icmF, tssC*::Tn5, *pvdL*::Tn5, and *pvdE*::Tn5 mutants through a fluorescence microscope with a DAPI filter. (Scale bar = 2 μm). (**b**) Quantification of periplasmic and cytoplasmic pyoverdine was conducted by measuring fluorescence at excitation 405 nm and emission 460 nm. Pyoverdine values were normalized against the cell optical densities (Ex405, Em 460/OD_600_), and relative values were determined by comparing each value to the periplasmic mean value of the wild-type. Values are mean ± SD of three independent experiments. *P. taiwanensis* and mutants (*clpV*::Tn5, Δ*icmF, tssC*::Tn5, *pvdL*::Tn5 and *pvdE*::Tn5) were grown on the iron-limited media for 12 h at 28 °C.

**Figure 6:**
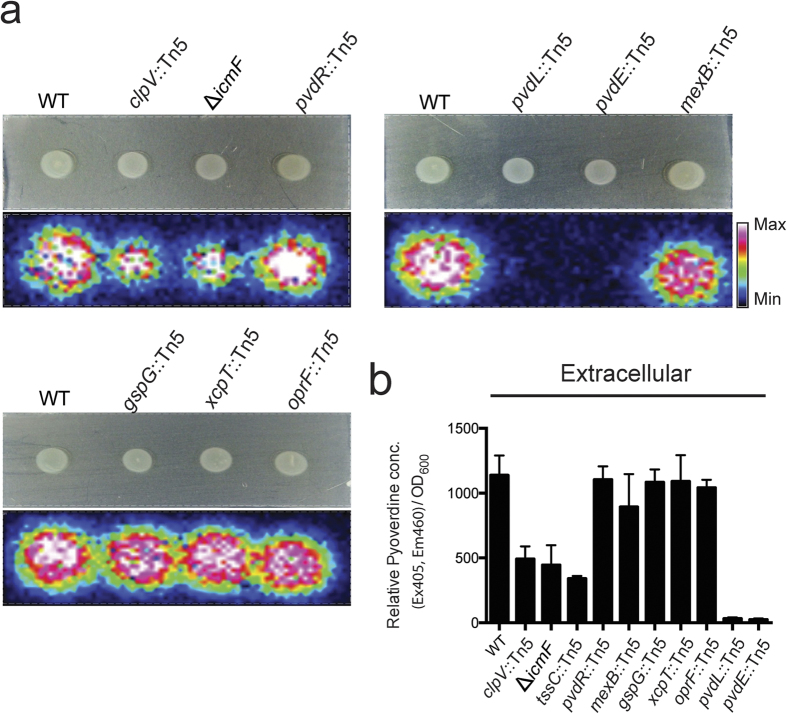
MALDI-IMS analysis and quantification of extracellular mature pyoverdine. (**a**) MALDI-IMS analysis of secretory mature pyoverdine (*m/z* 1044) on the surface of iron-limited agar plates of wild-type, T6SS *clpV*::Tn5, T6SS Δ*icmF,* T2SS *gspG*::Tn5, T2SS *xcpT*::Tn5, PvdRT-OmpQ *pvdR*::Tn5, MexAB-OprM *mexB*::Tn5, outer membrane protein Δ*oprF*::Tn5 mutants. Bacteria were grown for 12 h on the iron-limited agar plates. (**b**) Quantification of extracellular mature pyoverdine was detected by measuring fluorescence at excitation 405 nm and emission 460 nm. Pyoverdine values were normalized against the cell optical densities (Ex 405, Em 460/OD_600_), and relative values were determined by comparing each value to the periplasmic mean value of the wild-type. Values are mean ± SD of three independent experiments. Bacteria were grown on the iron-limited media for 12 h at 28 °C.

Next, the other component mutants of T6SS were used to confirm the involvement of T6SS in pyoverdine secretion. After screening 11,600 mutants, only one additional T6SS component mutant *tssC*::Tn5 (GenBank AC no. KU668948) was isolated. We further generated the third component mutant Δ*icmF* (GenBank AC no. KU668949) by site-specific recombination using the lambda-Red system. TssC is a T6SS core component that forms a contractile tail sheath^28^. IcmF is responsible for T6SS-mediated Hcp tube secretion^29^. We used live cell imaging to evaluate the involvement of T6SS in the secretion of mature pyoverdines from the periplasm to the extracellular medium. The DAPI filter set is appropriate for detection of the fluorescent pyoverdine. Interestingly, fluorescent pyoverdine was accumulated more in the periplasm of T6SS mutants (*clpV*::Tn5, *tssC*::Tn5 and Δ*icmF*) compared to WT under fluorescence microscopy with DAPI filter (Fig. 5a). Wild-type only displayed slight fluorescence in the periplasm. In the *pvdL*::Tn5 and *pvdE*::Tn5 mutants no fluorescent pyoverdine could be visualized under fluorescence microscopy with DAPI filter (Fig. 5a). The maxima fluorescence wavelength ranges of pyoverdine were confirmed at 360–410 nm of excitation and 450–480 nm of emission (Supplementary Fig. S6a). Pyoverdine mutant strain *pvdL*::Tn5 displayed no florescence intensity of emission (Supplementary Fig. S6b). Together with ultraviolet (UV) light detection (Supplementary Fig. S6), these results show that the only florescent substance produced in *P. taiwanensis* is a pyoverdine. Next, we quantified mature pyoverdine (fluorescent pyoverdine) in the periplasm and cytoplasm of the wild-type and the four mutants defective in anti-*Xoo* activity by measuring fluorescence at Ex/Em = 405/460 nm (Fig. 5b). In the three T6SS mutants (*clpV*::Tn5, *tssC*::Tn5 and Δ*icmF*), the amounts of mature pyoverdine in the periplasm were higher than that of wild-type. Mature pyoverdine was not detected in the cytoplasm of negative controls (*pvdL*::Tn5 and *pvdE*::Tn5 mutants) (Fig. 5b). Our data showed that T6SS is involved in the secretion of *de novo* synthesized pyoverdine. However, all T6SS mutants did have significant, albeit low, levels of pyoverdine in the extracellular media or agar plates (Fig. 2b–d, and Fig. 6) suggesting that an additional pathway might be involved in pyoverdine secretion.

### Characterization of the role of other exporters in pyoverdine secretion

It has been reported that the efflux pumps MexAB-OprM and PvdRT-OpmQ are involved in the secretion of newly synthesized pyoverdine^14^,^15^. However, Imperi *et al*. reached a different conclusion, reporting that MexAB-OprM and PvdRT-OpmQ do not function in pyoverdine secretion^16^. We re-examined the roles of these efflux systems in pyoverdine secretion by MALDI-IMS. IMS data showed that, compared to the wild-type, *mexB*::Tn5 and *pvdR*::Tn5, the component mutants of MexAB-OprM and PvdRT-OpmQ, respectively, had similar levels of mature pyoverdine (*m/z* 1044) secreted around the colonies on the surface of iron-limited agar plates (Fig. 6a). Furthermore, we investigated whether the type II secretion system (T2SS), which is a protein secretion machinery that removes substrates from the cell periplasm^30^, is involved in pyoverdine secretion. T2SS *gspG*::Tn5 and *xcpT*::Tn5 mutants have similar levels of secretory pyoverdine compared to the wild-type (Fig. 6a). The major outer membrane protein OprF of *Pseudomonas* has multiple functions, including maintaining cell shape, growth in a low-osmolarity environment, environmental sensing, virulence, and possible involvement in iron translocation^31^. However, *oprF*::Tn5 mutant did not affect pyoverdine secretion in iron-limited agar plates (Fig. 6a). In contrast, T6SS *clpV*::Tn5 and Δ*icmF* mutants had significantly reduced levels of secreted pyoverdine and *pvdL*::Tn5 and *pvdE*::Tn5 did not produce any mature pyoverdine (*m/z* 1044) on the surface of iron-limited agar plates (Fig. 6a). We quantified the amounts of secreted fluorescent pyoverdine in culture supernatants of wild-type and mutants grown under iron-limited media (Fig. 6b). Consistent with the IMS analyses, the results showed that the levels of mature pyoverdine in supernatants were significantly reduced in T6SS *clpV*::Tn5*, tssC*::Tn5 and Δ*icmF* mutants and none was detected in *pvdL*::Tn5 and *pvdE*::Tn5 mutants, while all other mutants have normal levels of secreted pyoverdine (Fig. 6b). The data also confirmed that PvdL and PvdE are involved in the biosynthesis and maturation of pyoverdine. Taken together, the results from Figs 2,4,5 and 6 suggest that T6SS does not affect intracellular pyoverdine production, but does affect the translocation of pyoverdine from the periplasm to the culture medium.

### Determination of P. taiwanensis pyoverdine toxicity against Xoo

**Figure 7:**
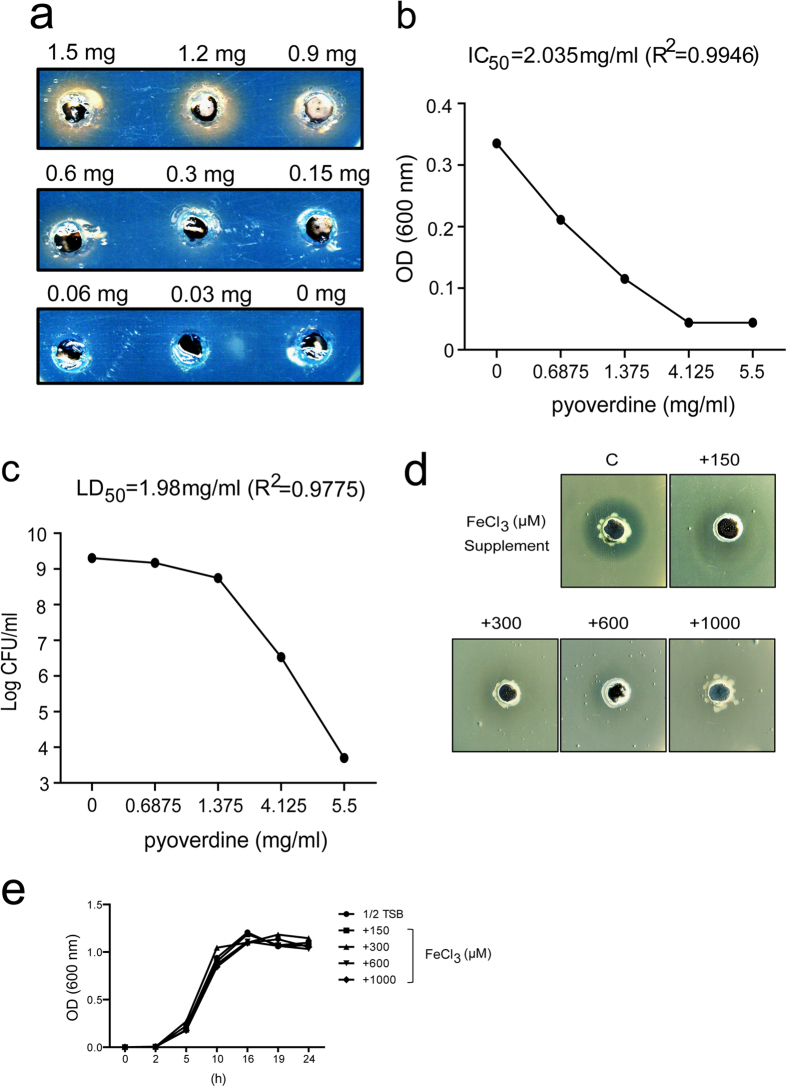
Pyoverdine activity assay. (**a**) Purified pyoverdine was loaded into the hole in CAS agar and incubated overnight at 28 °C. (**b**) Inhibitory concentration (IC_50_) and (**c**) lethal dose (LC_50_) of pyoverdine against *Xoo* were assayed by measuring absorbance at 600 nm and applying CFU, respectively. (**d**) Different concentrations of iron (150, 300, 600, 1000 μM FeCl_3_) were applied to antagonistic agar plates. (**e**) Cell growth of *P. taiwanensis* was detected in 1/2 TSB growth medium with 150, 300, 600 or 1000 μM FeCl_3_ or without FeCl_3_.

To demonstrate the anti-*Xoo* activity of pyoverdine from *P. taiwanensis*, different concentrations of purified pyoverdine were tested using CAS agar plate assays (Fig. 7a). After demonstrating pyoverdine activity, inhibition of cell growth (IC_50_) and lethal dose (LD_50_) against *Xoo* were determined. The IC_50_ of pyoverdine toward *Xoo* was about 2.035 mg/ml (R^2^ = 0.9946) (Fig. 7b); and LD_50_ was about 1.98 mg/ml (R^2^ = 0.9775) (Fig. 7c). The IC_50_ and LD_50_ data suggest that pyoverdine has anti-*Xoo* activity. Due to the high dose of pyoverdine needed to reach IC_50_ and LD_50_, we expect other factors to participate in anti-*Xoo* activity. However, pyoverdine is a key factor for anti-*Xoo* activity, since the pyoverdine synthesis *pvdL*::Tn5 mutant displayed no anti-*Xoo* activity (Fig. 2a).

To further clarify the role of pyoverdine in the antagonistic activity of *P. taiwanensis* against *Xoo*, iron-enriched culture media were used to examine pyoverdine activity. The culture broth of *P. taiwanensis* showed a dose-dependent decrease in toxicity when extra iron was applied to *Xoo-*containing plates (Fig. 7d). At higher concentrations of iron (300, 600, and 1000 μM FeCl_3_), *P. taiwanensis* had almost no antagonistic activity toward *Xoo* (Fig. 7d). The growth of *P. taiwanensis* was unaffected by the addition of iron compared to the control (1/2 TSB only) (Fig. 7e). However, we found that RNA expression levels of pyoverdine synthesis genes (*pvdL, pvdS, pvdI, pvdJ2*) and T6SS components (*clpV, icmF, vgrG, vasA, tssB*) were not significantly different under iron-enriched (LB) and iron-limited culture media (Supplementary Fig. S7). We propose that when there is a limited amount of iron in the environment, *P. taiwanensis* competes efficiently for iron by secreting pyoverdine to chelate iron and take up pyvoverdine-iron complexes through FpvA receptor, which results in retarded growth of *Xoo*. At higher concentrations of iron, however, the pyvoverdine secreted by *P. taiwanensis* is not sufficient to absorb all the available iron, which compromises its anti-*Xoo* activity.

## Discussion

**Figure 8:**
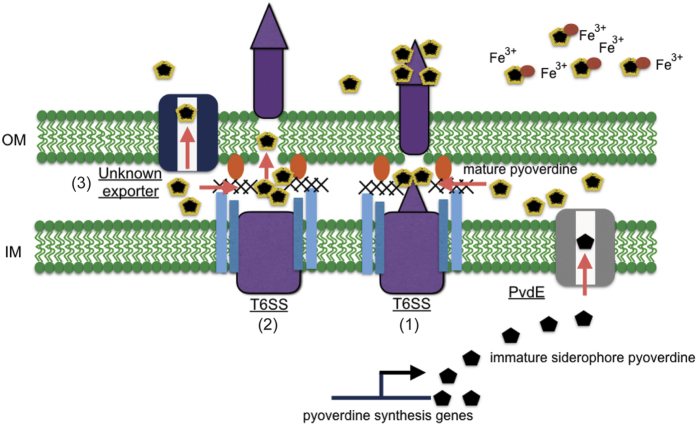
Schematic of the pyoverdine secretion pathways in *P. taiwanensis*. Pyoverdine precursors are synthesized in the cytoplasm and secreted into the periplasm by the inner membrane transporter PvdE, and then form mature fluorescent pyoverdine in the periplasmic space^7^,^44^,^45^. There are three possibilities that can explain the secretory pathways of pyoverdine: (1) pyoverdine adheres to T6SS tube structure and secretes into the culture medium after pushing the Hcp tube with the VgrG spike; (2) pyoverdine secretes into medium through passive diffusion after T6SS punches a hole in the outer-membrane; (3) pyoverdine is secreted via an unknown export system.

A schematic summary of pyoverdine transportation in *P. taiwanensis* is shown in Fig. 8. Our studies on the involvement of T6SS in the secretion of pyoverdine produced several interesting findings. Our results showed that T6SS is necessary for the secretion of wild-type levels of pyoverdine. However, the mechanism for the involvement of T6SS in the secretion of a small molecule remains to be elucidated. Based on the current results, there are three possible explanations of the secretory routes of pyoverdine: (1) pyoverdine adheres to the T6SS tube and secretes into culture medium after pushing the Hcp tube with the VgrG spike or via T6SS in a specific manner; (2) pyoverdine can be secreted into medium via passive diffusion during ejection of a T6SS Hcp-VgrG puncturing device; (3) secretion via an unknown export system. In addition to the above possibilities, we cannot exclude the possibility that T6SS is a signal in the regulation of pyoverdine secretion.

## Conclusion

Pyoverdine is a major siderophore in *Psedomonas taiwanensis* and acts as an antagonistic factor against rice bacterial blight pathogen *Xanthomonas oryzae* pv. *oryzae (Xoo*). We used MALDI-TOF imaging mass spectrometry (MALDI-IMS), which does not require extraction of molecules from the medium, to track secreted molecules from cell colonies and visualize their spatial distribution on the surface of agar plates. We showed that the type VI secretion system (T6SS) is necessary for the secretion of newly synthesized pyoverdine in *P. taiwanensis*. In addition to the T6SS, we expect that in the future additional exporters will be found to be involved in the secretion of endogenous pyoverdine. However, we have excluded MexAB-OprM and PvdRT-OpmQ efflux systems from involvement in the secretion of pyoverdine, despite them being previously suggested to be responsible.

## Methods

### Microorganisms and antagonistic assay

*Psedomonas taiwanensis* sp. nov. CMS^T^ (= BCRC17751^T^ = DSM 21245^T^) was isolated from soil and characterized using phenotypic and molecular taxonomic methods^32^. *Xanthomonas oryzae* pv. *oryzae (Xoo*) XF89b strain was isolated from rice blight disease in Taichung, Taiwan. *Pseudomonas syringae* pv. tomato (*Pst* DC3000) was provided by Laurent Zimmerli of the Institute of Plant Biology, National Taiwan University. *Pseudomonas putida* (BCRC 17059) and *Pseudomonas fluorescens* (BCRC 16016) were purchased from the Bioresource Collection and Research Center (BCRC, Taiwan).

Antagonistic assay of *P. taiwanensis* against *Xoo* was tested on 1/2 trypticase soy (TSB) agar plates (BD Biosciences) at 28 °C. *P. taiwanensis pre-culture* was grown in an iron-limited medium (M9 minimal *medium* supplemented with 1% *Casamino acids*, 1 mM MgSO_4_, and 0.5% glycerol) and incubated in a 500 mL flask containing 100 mL medium at 28 °C and 200 rpm for 24 h. *Xoo pre-culture* grown in 1/2 TSB medium was incubated at 28 °C for 1 day. *Xoo* was mixed with melted 1/2 TSB agar medium before being poured into an empty plate for competition assays. For bioassays, bacteria culture broth (10^9^ CFU/mL) or filter (0.22 μm) supernatant was injected (50 μL) into the hole of the *Xoo-*seeded 1/2 TSB agar plate until the inhibition zones had been characterized. The bacteria pellets were washed three times with PBS at 4 °C and resuspended in PBS. Cell density and cell viability were determined using optical density at OD_600_ and by counting CFU/ml. For the field trial, the leaves of the japonica rice Tainung 67 (TN 67) and the indica rice Taichung Sen 10 (TCS 10) were infected with *Xoo* by using scissors dipped in *Xoo* (1 × 10^9^ CFU/mL). After 1 week, we sprayed the rice with different dilutions of total broth (1 × 10^9^ CFU/mL) or cell-free supernatant (from 1 × 10^9^ CFU/mL broth) of *P. taiwanensis*. After 3 weeks, the symptoms of bacterial blight were recorded.

### Comparison of pyoverdine (m/z 1044) levels by LC/MS

After incubation for 1 day, the culture supernatants were collected by centrifugation for 10 min at 4500× g. The culture supernatants were sterilized through a 0.22-μm filter. A 10 mL aliquot of each filtered supernatant was dried by freeze drying and resuspended in 50% methanol. The total number of metabolites was detected using high-resolution liquid chromatography-mass spectrometry (LC/MS) (ESI-Orbitrap, conducted by the Metabolomics Core Facility, Academia Sinica, Taiwan). The peak height and area were determined for calculation of the pyoverdine level in LC/MS analyses.

### Construction of the transposon library

An EZ-Tn5 transposon mutagenesis kit (KAN-2; Epicentre) was used to make a random mutant library. EZ-Tn5 transposon mutagenesis was performed according to the manufacturer’s instructions. *P. taiwanensis* competent cells were prepared according to the method outlined in Choi *et al*.^33^. To screen the Tn5 mutant library, we utilized the *P. taiwanensis* mutagenesis library to incubate with *Xoo*, providing the opportunity to find virulence-related genes. The flanking sequences of insertion sites were amplified by TAIL-PCR^34^. Two sets of random primers and the specific regions of the two ends of the transposon primers were designed by Sun *et al*.^35^. The Tn5 mutant strains of this study were further determined by PCR and sequencing. The mutant strains (*clpV*::Tn5, *pvdL*::Tn5, *pvdE*::Tn5) were determined by UV light (Supplementary Fig. S8). Except T6SS Δ*icmF* mutant, all mutants were generated by the EZ-Tn5 transposon system. We used the lambda Red recombinase system to generate Δ*icmF* mutant in *P. taiwanensis.* Manipulation of the lambda Red recombinase system was performed using a published protocol^36^

Southern blot analysis was used to check the number of Tn5 insertions in the mutants (Supplementary Fig. S9). NcoI- and EagI-digested genomic DNA of Tn5-inserted mutants were analyzed by Southern blot hybridization with a DIG-labelled PCR probe. The coding sequence of the kanamycin resistance gene was used as a probe to confirm the insertion number. After hybridization, the blots were developed using a detection kit (Roche). We confirmed that EZ-Tn5 transposon system only generated progenies with one copy of *Kan* cassette insertion.

In order to monitor downstream gene expression of *clpV,* we compared PT3445 and *yhfE* gene expression in the WT and *clpV* mutant using RT-PCR. The result showed that *clpV*::Tn5 mutation does not affect downstream gene expression (Supplementary Fig. S10). The *clpV*::Tn5 mutant was complemented by expression of a wild-type *clpV* gene using the broad host range vector pCPP30 37. Induction of pCPP30 harboring *clpV* fragment was performed overnight by adding 1 mM isopropyl-β-D- thiogalactopyranoside (IPTG) to iron-limited medium.

### Secretory T6SS component

VgrG was detected in the culture supernatant by western blotting using anti-*Agrobacterium tumefaciens* VgrG antibody^38^. The RNA polymerase α-subunit RpoA, which was used as a loading control in western blots, was detected using anti-*Agrobacterium tumefaciens* RpoA antibody^38^. Both anti-VgrG and anti-RpoA antibodies were provided by Dr. Erh-Min Lai, Institute of Plant and Microbial Biology, Academia Sinica, Taiwan. Twenty-four hour culture of *P. taiwanensis* wild-type and *clpV* mutant in iron-limited medium were grown to an optical density at 600 nm (OD_600_) of ~0.8. After centrifugation, at 4500× g for 10 min, the culture supernatant was sterilized through 0.22 μm Durapore polyvinylidene fluoride (PVDF) (lowest protein binding) syringe filters. Cell-free culture supernatant proteins (20 ml) were precipitated by adding trichloroacetic acid (TCA) to final 10% TCA concentration overnight at 4 °C and the pellet was washed twice with ice-cold acetone to remove residual TCA. TCA-precipitated secretory proteins were dissolved in 9.8 M urea solution.

### MALDI-IMS

Comparison of the distribution of metabolites on the surface of competition agar plates using MALDI-IMS revealed interesting differences in the ions secreted by the wild type and mutants of *P. taiwanensis*. The regions of interest of the bacterial colonies were excised, and placed on glass slides. Slides with interesting target samples were covered with a thin layer of universal MALDI matrix (Sigma-Aldrich) deposited over the sample using a 50 μm sieve. The matrix-covered agar samples were dehydrated in an incubator at 37 °C overnight prior to IMS. The samples were analyzed by a Bruker Autoflex Speed MALDI-TOF/TOF MS and the data were collected. Samples were analyzed in positive reflectron ion mode, screened at 200 μm laser intervals with the acquisition mass range set at 100–2000 Da. The equipment was calibrated using a standard peptide calibration mixture (Peptide Calibration Standard 206195, Bruker, 1000–3200 Da) and matrix. The IMS data were analyzed using Fleximaging 3.0 software (Bruker). The intensity of molecules was presented as gradient colors.

### Purification and determination of pyoverdine

The method for pyoverdine purification was modified from Yin *et al*.^39^. Fifty mL of *P. taiwanensis* in a 250 mL flask was incubated in iron-limited medium at 28 °C and 200 rpm for 24 h. The culture supernatant was collected by centrifugation at 4,600× g for 15 min at 4 °C and filtered through 0.22 μm sterile low protein binding polyvinylidene fluoride (PVDF) membrane filters (Millex-GV; Millipore). A chelating Cu-sepharose column was used to purify pyoverdine^40^. Copper ions (Cu^2+^) were used for recharging the sepharose from Ni-sepharose High Performance media (GE). Five milliliters of Ni-sepharose was loaded in a 0.8 × 4 cm Poly-Prep chromatography column (Bio-Rad) and buffer was allowed to flow through using a gravimetric method. To remove the residual Ni^2+^, the Ni-sepharose column was washed with five column volumes of buffer (0.02 M Na_2_HPO_4_, 0.5 M NaCl, and 0.05 M EDTA; pH 7.2). Then the column was washed to remove residual EDTA with at least five column volumes of distilled water and the sepharose was recharged with 0.5 mL of 1 M CuSO_4_. Then, the Cu-sepharose was washed with five column volumes of binding buffer (0.02 M Na_2_HPO_4_, 1 M NaCl; pH 7.2).

The filtered culture supernatant was mixed with the binding buffer at a ratio of 1:1. Twenty milliliters of mixture was loaded onto a Cu-sepharose column to purify pyoverdine or other siderophores. Then, the column was washed with five column volumes of binding buffer again. Finally, the siderophores were eluted by elution buffer (0.02 M Na_2_HPO_4_ and 1M NH_4_Cl; pH 7.2) and dried in a Freeze Dryer (FD12-24P-L-80, Kingmech, Taiwan). The purified compounds were checked by HPLC analysis with an RP-Amide C16 column (4.6 × 250 mm, 5 μm; Sigma-Aldrich) and MALDI-TOF MS. The absorption maxima wavelength of fluorescent pyoverdine was evident at 370–410 nm (excitation) and 450–470 nm (emission) using a Tecan Infinite M1000 pro. (The chromatography of HPLC was monitored over a range of 200–500 nm by a UV absorption detector.) The acetonitrile-water gradient of the HPLC mobile phase was from 50% to 0% acetonitrile over 10 min at a flow rate of 1 mL/min. Fractions were collected every minute and monitored by MALDI-TOF. ESI-Orbitrap (conducted by the Metabolomics Core Facility, Academia Sinica, Taiwan) was used to collect tandem mass spectra for the structural elucidation of pyoverdine.

For observing subcellular location of mature pyoverdine in live cells, *P. taiwanensis* wild-type and mutants were detected using an Olympus BX61 microscope equipped with a DAPI filter. The DAPI filter set is appropriate for detection of the fluorescent pyoverdine. The absorption wavelength range of fluorescent pyoverdine was measured at 320–440 nm (excitation) and 440–520 nm (emission) using a Tecan Infinite M1000 pro (Supplementary Fig. S5a).

### Inhibitory concentration (IC_50_) and lethal dosage (LD_50_) assays

Purified pyoverdine was dissolved in 1/2 TSB and sterilized by a 0.22 μm filter. 1/2 TSB media containing pure pyoverdine from 5.5 to 0 mg/ml was placed in tubes containing 2 ml of 1/2 TSB. To study the effect of pyoverdine on growth of *Xoo*, absorbance at 600 nm and the number of viable cells (cfu/ml) were assayed after incubation for two nights at 28 °C and 200 rpm. Assays were conducted in triplicate and consistent results were obtained.

### CAS plate assay

Chrome azurol S (CAS) was developed by Schwyn and Neilands to determine siderophore activity^41^. It is a universal method that detects the mobilization of iron, thus assaying siderphore production. To prepare 100 ml CAS dye, 60.5 mg CAS powder (Sigma) was dissolved in 50 ml distilled water and mixed with 10 ml of 1 mM iron solution (anhydration FeCl_3,_ Alfa Aesar). Then, 40 ml of 72.9 mg HDTMA (Sigma) was added slowly to 60 ml CAS solution with FeCl_3_ and autoclaved to sterilize. After the CAS cooled down and could be hand-held, one-tenth of the CAS solution was mixed with LP agar medium and immediately poured into plates.

CAS plates were used to demonstrate the activity of purified pyoverdine. Different concentrations of purified pyoverdine were injected into the hole (5 mm) of CAS plates. Plates were incubated at 28 °C for 6 h or until they had a yellow halo appearance.

### Quantification of Subcellular Pyoverdine

Extracellular pyoverdine was quantified from cell free culture supernatant of *P. taiwanensis* after growing in an iron-limited medium for 12 h. Culture supernatant was collected by centrifugation (6,000 × g, 3 min) and filtered by a 0.22 μm pore size filter. To separate the periplasmic and cytosolic fractions, spheroplasts were obtained according to the method outlined in Imperi *et al*.^42^. Cell pellets (3 × 10^9^ cells) were washed three times in PBS buffer (pH 7.4). The cell pellets were suspended in 1 mL of the spheroplasting buffer (10 mM Tris-HCl, pH 8.0, 200 mM MgCl_2_, 0.5 mg/mL lysozyme), and incubated with gentle shaking for 30 min at room temperature. After incubation, the periplasmic fractions were collected by centrifugation (11,000 × g, 15 min, 4 °C). The spheroplasts were washed three times in PBS buffer (pH 7.4). The pellets were suspended in 1 mL of sonication buffer (10 mM Tris-HCl, pH 8.0, 100 mM NaCl) and lysed by sonication. After centrifugation (16,000 × g, 5 min), cell debris was removed to obtain the cytoplasmic fractions. Mature fluorescent pyoverdine was determined using appropriate dilutions of dilution buffer (100 mM Tris-HCl) using a fluorescence Plate Reader (Victor 2, Perkin-Elmer) with excitation/emission wavelengths of 405/460 nm. Pyoverdine values were normalized against the cell optical density (OD_600_).

### Real-time PCR assay

Total RNA was extracted using total RNA Miniprep Purification Kit (Genemark). Five micrograms of total RNA was reverse transcribed with M-MLV reverse transcriptase (Invitrogen) and random hexamers. Quantitative real-time PCR was performed on an ABI PRISM 7500 Sequence Detection System using Power SYBR Green PCR Master Mix (Applied Biosystems). The 16S rRNA gene was used as an internal control for real-time PCR assay. Each 20 μl real-time PCR reaction volume contained 10 ng cDNA, 1 × SYBR Green Mix, and 5 μM of the forward and reverse primers. Each experiment was conducted three times.

## Additional Information

**How to cite this article**: Chen, W.-J. *et al*. Involvement of type VI secretion system in secretion of iron chelator pyoverdine in *Pseudomonas taiwanensis. Sci. Rep.* **6**, 32950; doi: 10.1038/srep32950 (2016).

## Supplementary Material

Supplementary Information
