# Supplementary material for: Involvement of type VI secretion system in secretion of iron chelator pyoverdine in Pseudomonas taiwanensis
Source: Sci Rep. 2016 Sep 8;6:32950. doi: 10.1038/srep32950 (PMC5015096; doi:10.1038/srep32950)
Supplement: Supplementary Information [file srep32950-s1.pdf]

Supplementary data for

Involvement of Type VI secretion system in secretion of iron chelator pyoverdine in *Pseudomonas taiwanensis*

Wen-Jen Chen[1,2], Tzu-Yen Kuo[1] , Feng-Chia Hsieh[3], Pi-Yu Chen[1], Chang-Sheng Wang[4], Yu-Ling Shih[5], Ying-Mi Lai[1], Je-Ruei Liu[1,2], Yu-Liang Yang[1], Ming-Che Shih[1,2*]*

[1]Agricultural Biotechnology Research Center, Academia Sinica, Taipei, 11529, Taiwan

[2]Institute of Biotechnology, National Taiwan University, Taipei, 10617, Taiwan

[3]Biopesticide Division, Taiwan Agricultural Chemicals and Toxic Substances Research Institute, Council of Agriculture, Taichung, 41358, Taiwan

[4]Department of Agronomy, National Chung Hsing University, Taichung, 40227, Taiwan

[5]Institute of Biological Chemistry, Academia Sinica, Taipei, 11529, Taiwan

* To whom the correspondence should be addressed.

This word file includes:
Materials and Methods
Figures S1 to S10
Tables S1 and S2

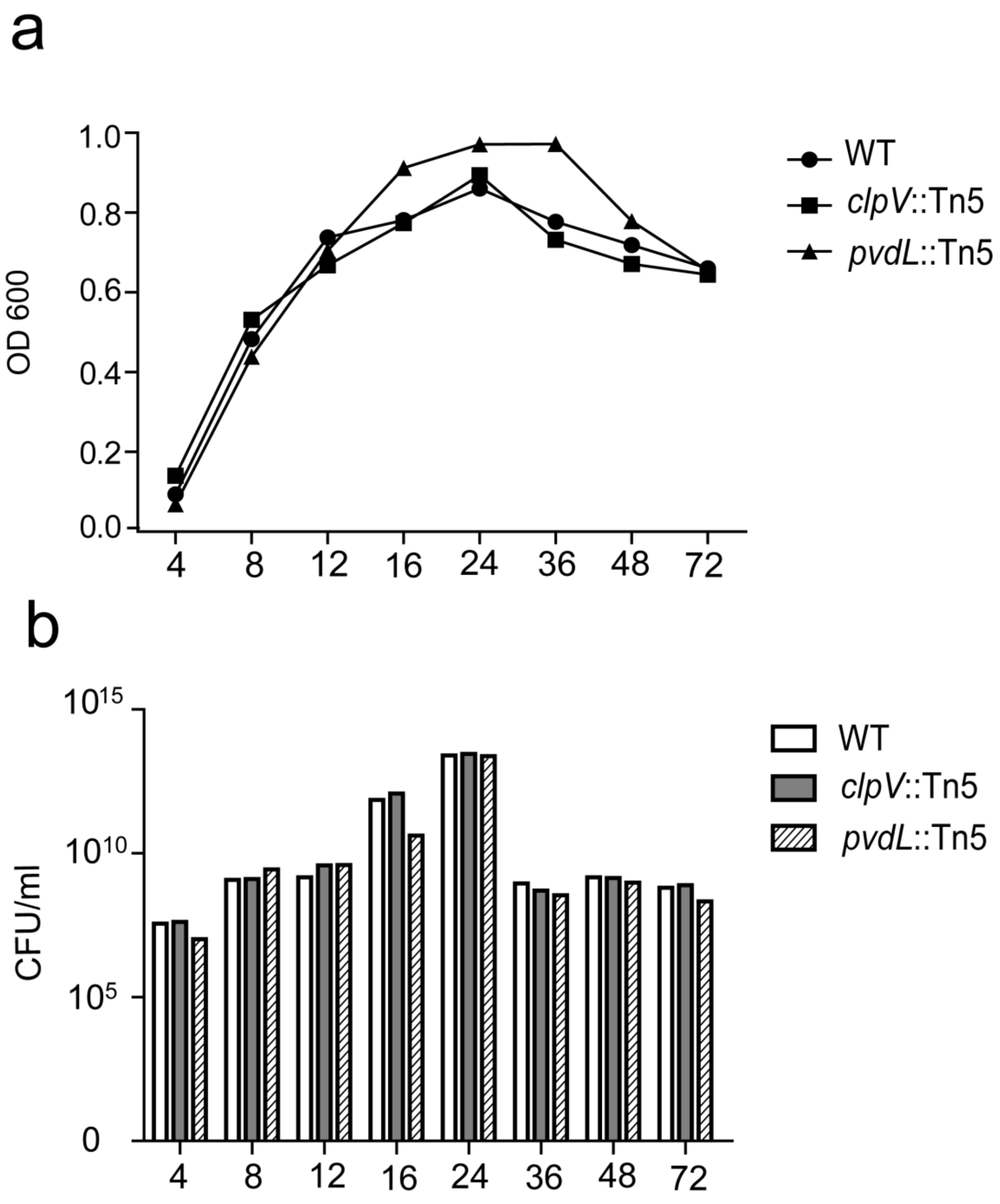

Supplementary Figure 1. Comparison of the growth of *P. taiwanensis* wild type (WT), T6SS mutant (*clpV*::Tn5), and Pyoverdine deficient muatnt (*pvdL*::Tn5) in iron-limited medium were measured by (a) cell density at OD_{600} and (b) colony forming units (CFU).

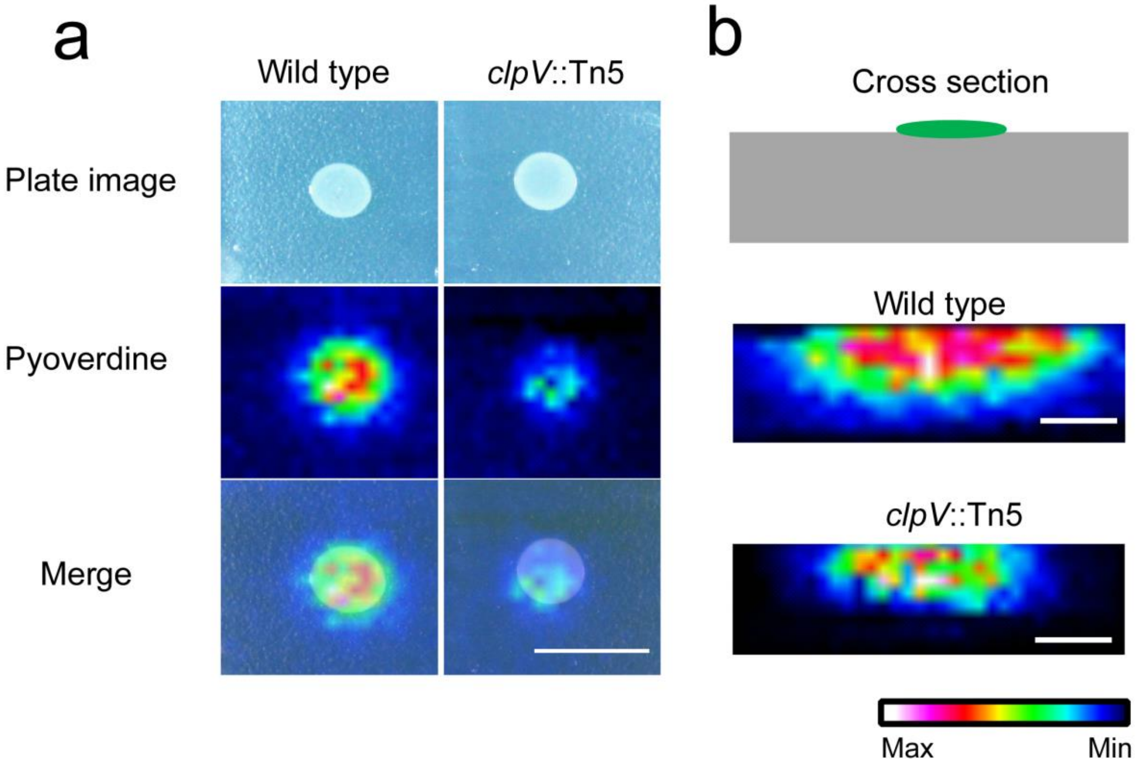

Supplementary Figure 2. MALDI-IMS image of pyoverdine from wild type and *clpV*::Tn5 mutant (**a**) Pyoverdine accumulated on the surface of the wild type and *clpV* mutant was compared after incubation for 16 h or (**b**) on a vertical section of iron-limited agar plates after incubation for 48 h by IMS. (**a**) Scale bar, 10 mm. (**b**) Scale bar, 5 mm. Intensity gradients for pyoverdine are illustrated by color histograms (maximum, white; minimum, black).

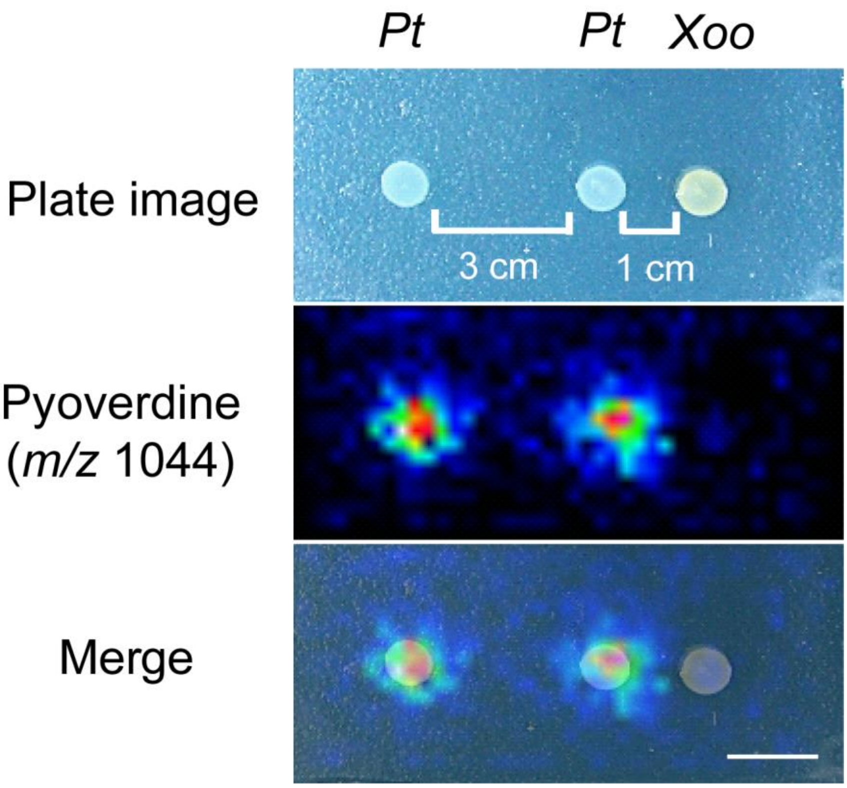

Supplementary Figure 3. Pyoverdine is not stimulated by *Xoo*.

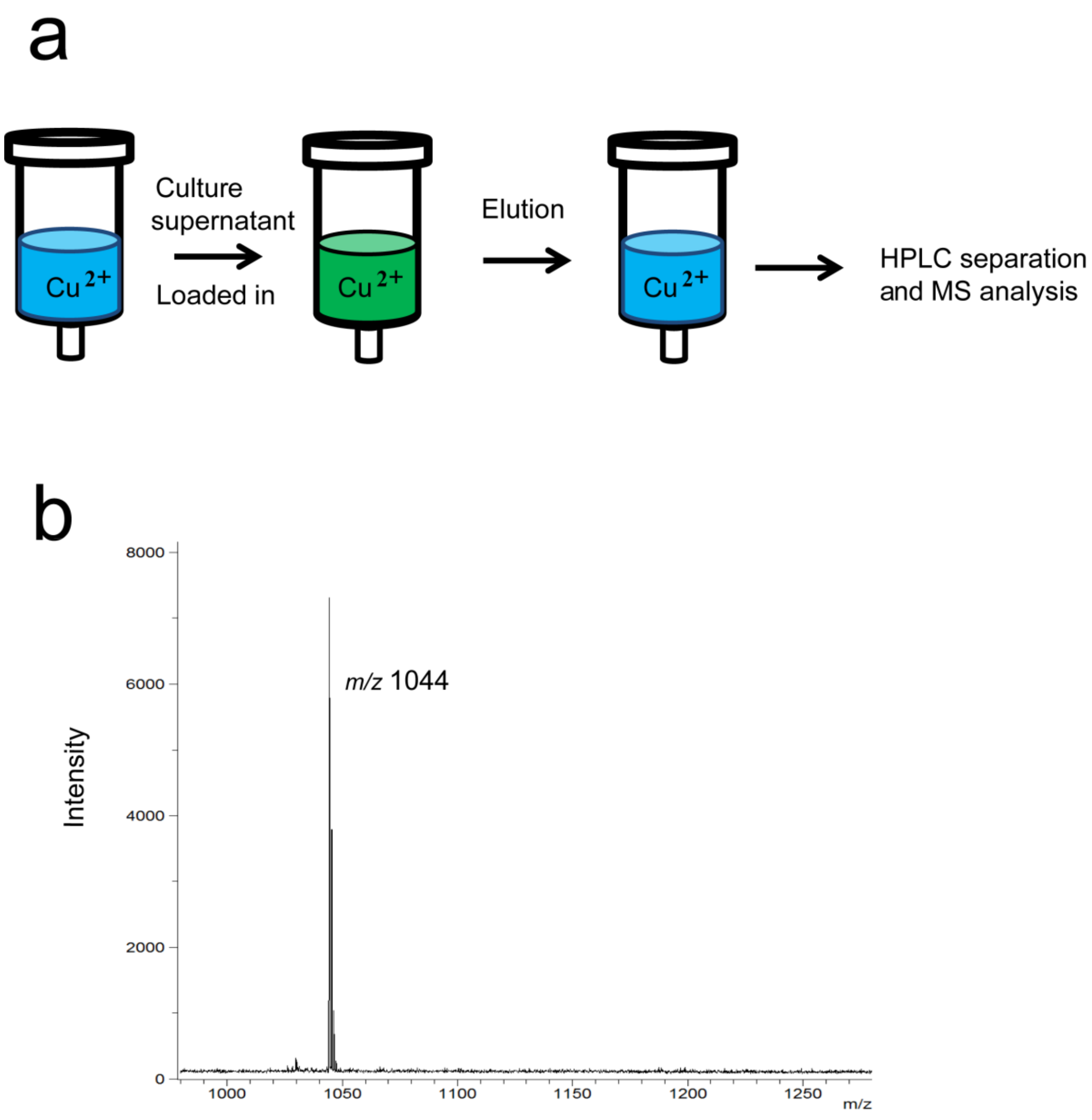

Supplementary Figure 4. Purification of pyoverdine by Cu-sepharose and HPLC, and determined by MALDI-TOF MS analysis.

a

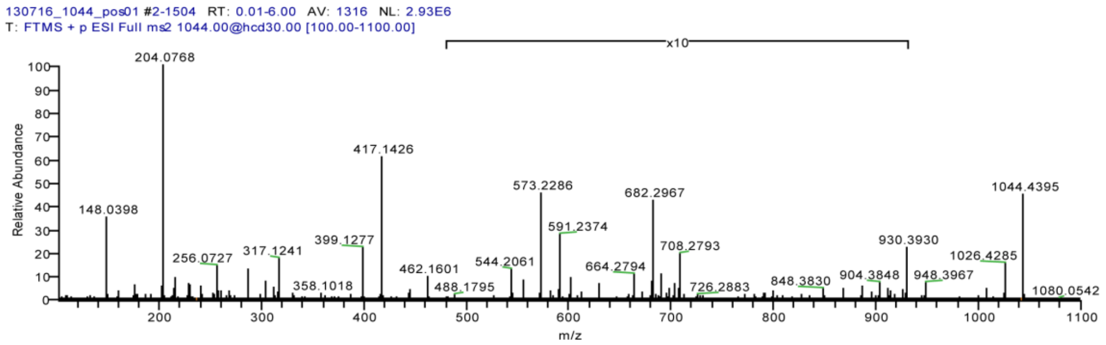

b

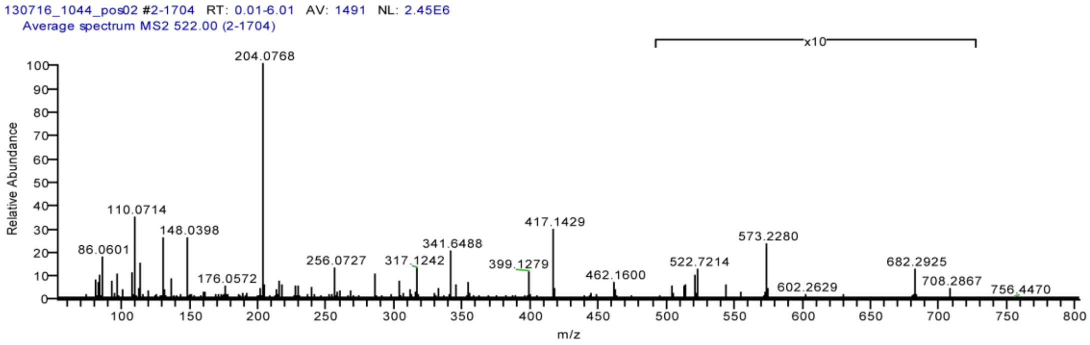

Supplementary Figure 5. MS/MS spectral analysis of pyoverdine.

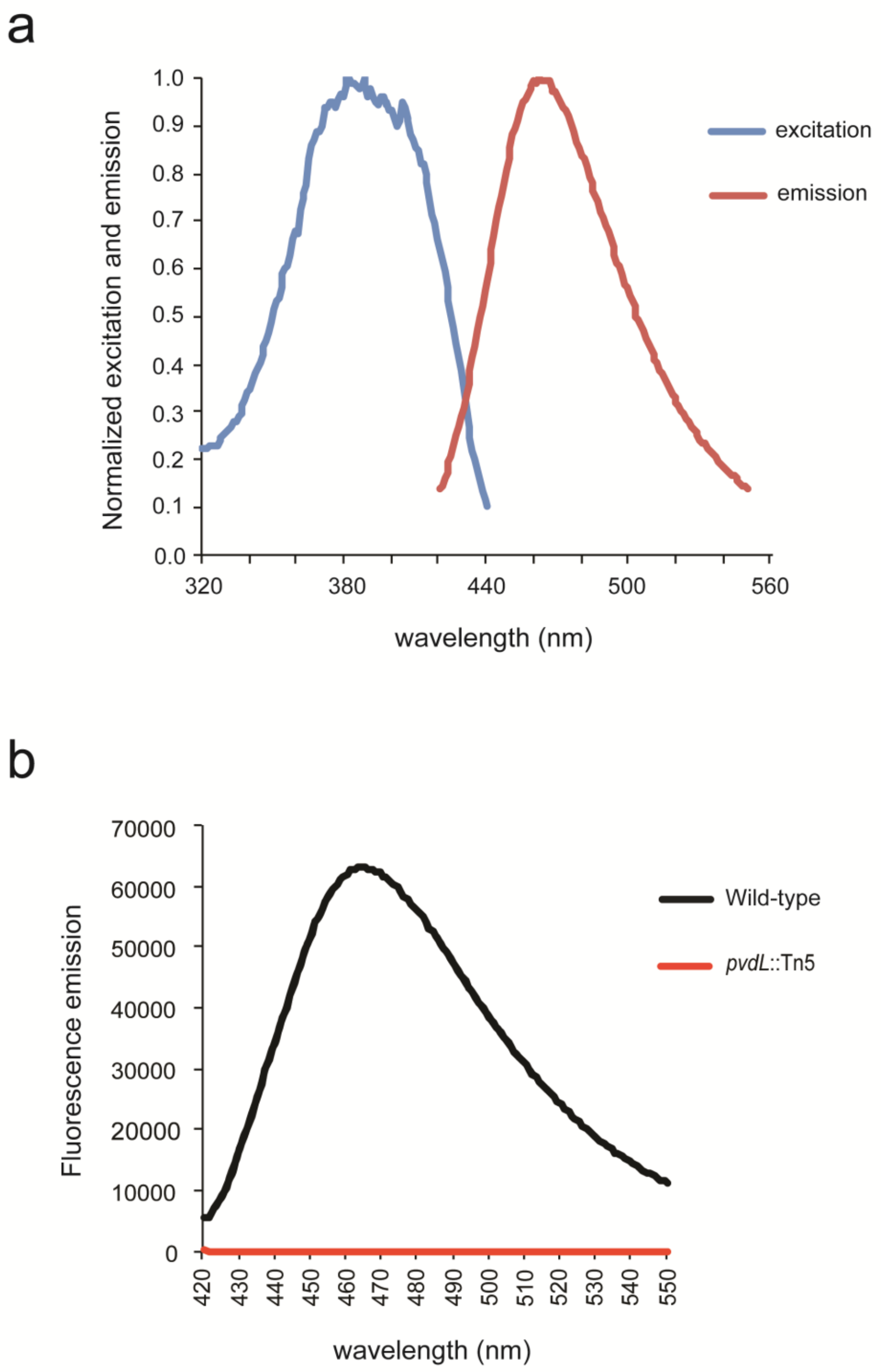

Supplementary Figure 6. Detection of maxima excitation and emission wavelengths. (**a**) Wavelength scanning was performed from 320 nm to 440 nm (Ex) and 420-550 nm (Em) to determine the excitation and emission ranges for purified pyoveridine (100 µg/ml) in 50% methanol using a Tecan Infinite M1000 pro. (**b**) Fluorescence intensity was detected between wild-type and pyoverdine *pvdL*::Tn5 mutant on excitation at 405 nm and emission at 420-550 nm.

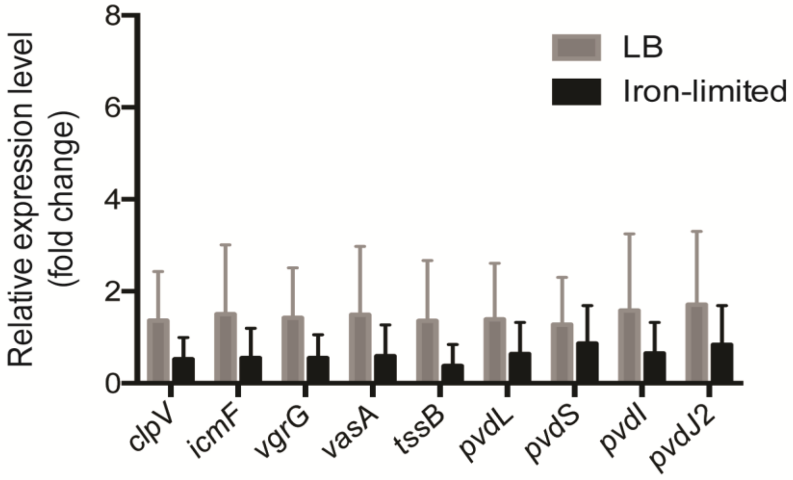

Supplementary Figure 7. Relative expression levels of T6SS components and pyoverdine synthesis genes. The T6SS components (*clpV*, *icmF*, *vgrG*, *vasA*, *tssB*) and pyoverdine synthesis genes (*pvdL*, *pvdS*, *pvdI*, *pvdJ2*) expression levels of *P. taiwanensis* were analyzed by real-time PCR and compared with internal control 16S rRNA. *P. taiwanensis* was collected after 24 hours incubation under LB or iron-limited culture medium.

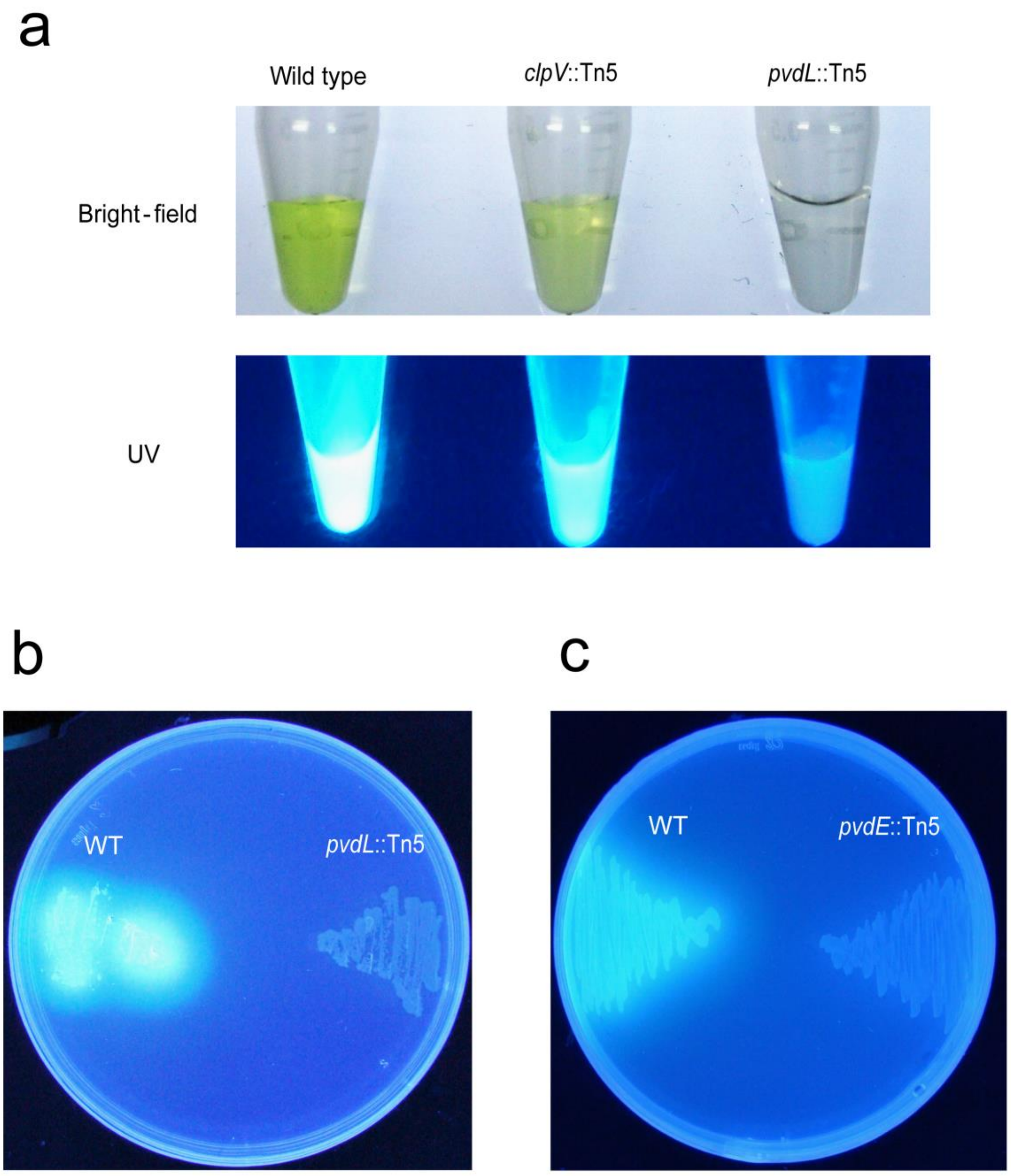

Supplementary Figure 8. Pyoverdine was detected in *clpV*::Tn5, *pvdL*::Tn5, and *pvdE*::Tn5 mutants by UV. Characterization of secreted pyoverdine in culture supernatant (**a**) and agar plates (**b**).

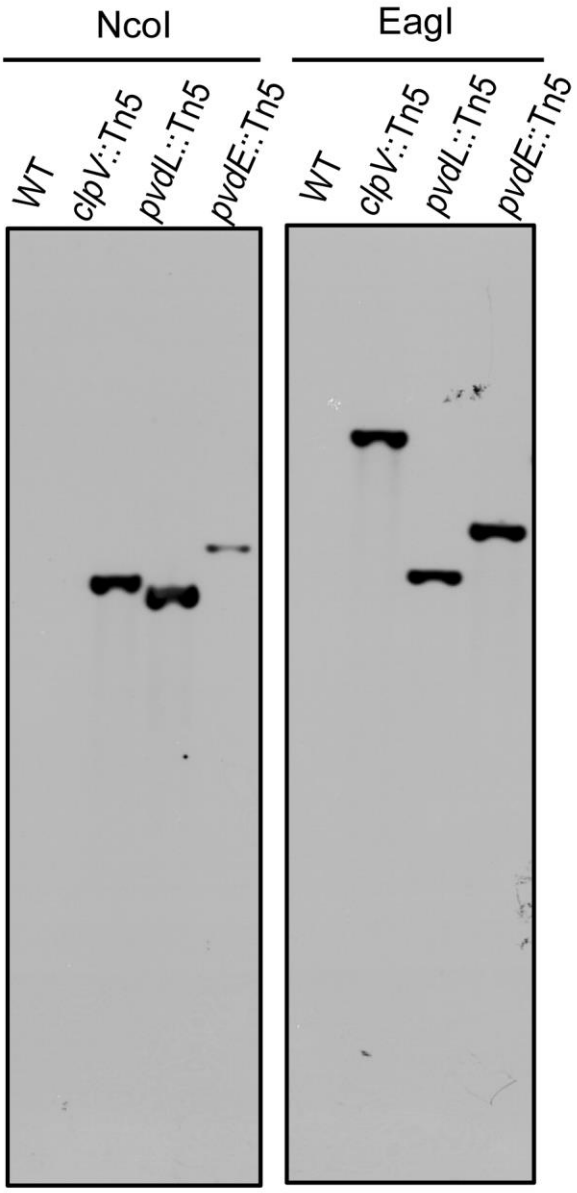

Supplementary Figure 9. Southern blot analysis of NcoI and EagI-digested DNA hybridized with the DIG-labelled Tn5 transposon fragment. After identification by TAIL-PCR and sequencing, genomic DNA was extracted from *clpV*::Tn5, *pvdL*::Tn5, and *pvdE*::Tn5 mutants. Genomic DNA (10 µg) was digested with NcoI and EagI. NcoI and EagI digested DNA were electrophoresed and transferred to a nylon membrane, and then hybridized with a Dig-labeled kanamycin probe to examine the copy number of Tn5 transposon.

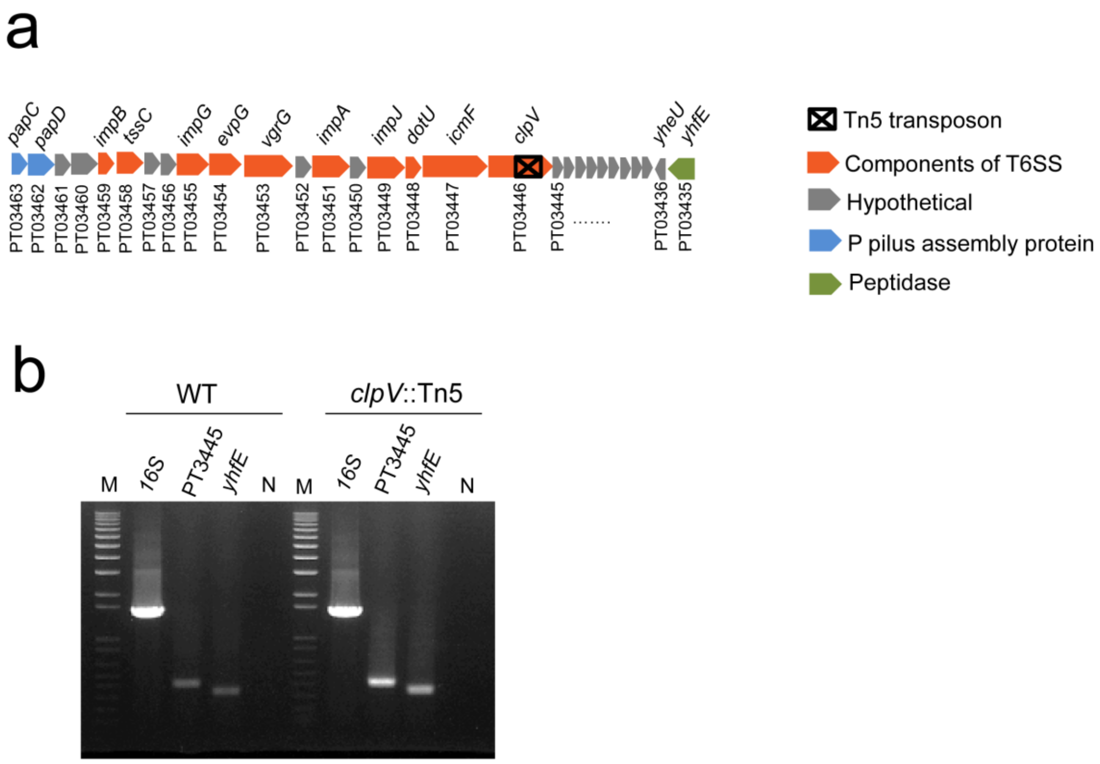

Supplementary Figure 10. Expression of downstream genes in the Tn5 inserted-*clpV* mutant. Expression of the PT3445 and *yhfE* genes was analyzed in the wild type and *clpV*::Tn5 by RT-PCR.

Table S1. FTMS2 annotation (Da) of peptide 1044

b			***b*-H_2O**			***b*+H_2O**			***a***			**Residues**	***y*+2H**		
Calcd.	Found	Error	Calcd.	Found	Error	Calcd.	Found	Error	Calcd.	Found	Error		Calcd.	Found	Error
358.1039	358.1046	0.0007	340.0933	340.0924	-0.0009	376.1145	376.1155	0.0010				**Chromophore**			
445.1359	445.1359	0	427.1253	427.1243	-0.0010	463.1465	463.1473	0.0008	417.1410	417.1426	0.0016	**Ser**			
573.2309	573.2286	-0.0023	555.2203	555.2196	-0.0007	591.2415	591.2434	0.0019				**Lys**	600.3106	600.3079	-0.0027
726.2847	726.2883	0.0036	708.2741	708.2716	-0.0025	744.2953	744.2977	0.0024	698.2898	698.2926	0.0028	**OH-His**	472.2156	472.2138	-0.0018
827.3324	827.3371	0.0047	809.3218	809.3261	0.0043				799.3375	799.3357	-0.0018	**Thr**	319.1618	319.1633	0.0015
914.3644	914.3653	0.0009	896.3538	896.3538	0				886.3695	886.3718	0.0023	**Ser**	218.1141	218.1137	-0.0004
												**OH-Orn**	131.0821	131.0816	-0.0005

Table S2. Summary of *P. taiwanensis* pyoverdine loci genes

No.[a]	Gene	Function	NCBI GenBank nb.	Seq identity[b]
1	*pvdL*	chromophore peptide synthetase	KM036007	72.62% (*Pseudomonas fluorescens* F113)
2	*pvdS*	sigma factor	KM036008	89.53% (*Pseudomonas aeruginosa* PAO1)
3	*pvdYII*	probable transcriptional regulator	KM036009	72.13% (*Pseudomonas aeruginosa* DK2)
4	*pvdQ*	acyl-homoserine lactone acylase	KM036010	75.33% (*Pseudomonas putida* NBRC 14164)
5	sleB	cell wall hydrolase SleB	KM036011	86.70% (*Pseudomonas putida* H8234)
6		auxin efflux carrier	KM036012	92.46% (*Pseudomonas putida* W619)
7		hypothetical protein	KM036013	69.39% (*Pseudomonas putida* GB-1)
8		LysR family transcriptional regulator	KM036014	94.43% (*Pseudomonas putida* H8234)
9		metal ABC transporter substrate-binding protein	KM036015	81.72% (*Pseudomonas putida* H8234)
10		ABC transporter	KM036016	87.21% (*Pseudomonas fluorescens* F113)
11		ABC transporter-like protein	KM036017	93.15% (*Pseudomonas putida* GB-1)
12		cation ABC transporter periplasmic cation-binding protein	KM036018	95.34% (*Pseudomonas entomophila* L48)
13		hypothetical protein	KM036019	77.78% (Pseudomonas putida GB-1)
14		hypothetical protein	KM036020	89.52% (*Pseudomonas entomophila* L48)
15		thiamine pyrophosphate-requiring enzyme-like protein	KM036021	65.12% (*Pseudomonas putida* H8234)
16		peptidase	KM036022	75.26% (*Pseudomonas putida* H8234)
17	*pvdI*	Non-ribosomal peptide synthetase	KM036023	63.64 %(*Pseudomonas sp.* UW4)
18	*syrP*	pyoverdine biosynthesis regulatory protein	KM036024	79.06% (*Pseudomonas sp.* UW4)
19	*pvdJ*	Non-ribosomal peptide synthetase	KM036025	55.64% (*Pseudomonas sp.* UW4)
20	*pvdJ* (2)	Non-ribosomal peptide synthetase	KM036026	66.08% (*Pseudomonas sp.* UW4)
21	*lip2*	lipase	KM036027	67.81% (*Pseudomonas sp.* UW4)
22	*fpvA*	TonB-dependent siderophore receptor	KM036028	66.34% (*Pseudomonas sp.* UW4)
23	*pvdE*	pyoverdine biosynthesis protein	KM036029	67.10% (*Pseudomonas aeruginosa* DK2)
24	*pvdA*	L-ornithine N5-oxygenase	KM036030	84.39% (*Pseudomonas entomophila* L48)
25	*pvdO*	No known function	KM036031	71.27% (*Pseudomonas aeruginosa* M18)
26	*pvdN*	aminotransferase	KM036032	65.58% (*Pseudomonas putida* NBRC 14164)
27	*pvdM*	dipeptidase	KM036033	78.20% (*Pseudomonas fluorescens* F113)
28		hypothetical protein	KM036034	69.66% (*Pseudomonas putida* W619)
29	*pbpG*	D-alanyl-D-alanine endopeptidase	KM036035	94.56% (*Pseudomonas putida* W619)

(a) The genomic organization of pyoverdine loci genes in *P. taiwanensis* against *Pseudomonas* database

(b) Percentage similarity between *P. taiwanensis* amino acid sequence and the alignment among *Pseudomonas* database.
